# SlideToolkit: An Assistive Toolset for the Histological Quantification of Whole Slide Images

**DOI:** 10.1371/journal.pone.0110289

**Published:** 2014-11-05

**Authors:** Bastiaan G. L. Nelissen, Joost A. van Herwaarden, Frans L. Moll, Paul J. van Diest, Gerard Pasterkamp

**Affiliations:** 1 Department of Vascular Surgery, University Medical Center Utrecht, Utrecht, The Netherlands; 2 Department of Pathology, University Medical Center Utrecht, Utrecht, The Netherlands; 3 Laboratory of Experimental Cardiology, University Medical Center Utrecht, Utrecht, The Netherlands; University of Torino, Italy

## Abstract

The demand for accurate and reproducible phenotyping of a disease trait increases with the rising number of biobanks and genome wide association studies. Detailed analysis of histology is a powerful way of phenotyping human tissues. Nonetheless, purely visual assessment of histological slides is time-consuming and liable to sampling variation and optical illusions and thereby observer variation, and external validation may be cumbersome. Therefore, within our own biobank, computerized quantification of digitized histological slides is often preferred as a more precise and reproducible, and sometimes more sensitive approach. Relatively few free toolkits are, however, available for fully digitized microscopic slides, usually known as whole slides images. In order to comply with this need, we developed the slideToolkit as a fast method to handle large quantities of low contrast whole slides images using advanced cell detecting algorithms. The slideToolkit has been developed for modern personal computers and high-performance clusters (HPCs) and is available as an open-source project on github.com. We here illustrate the power of slideToolkit by a repeated measurement of 303 digital slides containing CD3 stained (DAB) abdominal aortic aneurysm tissue from a tissue biobank. Our workflow consists of four consecutive steps. In the first step (acquisition), whole slide images are collected and converted to TIFF files. In the second step (preparation), files are organized. The third step (tiles), creates multiple manageable tiles to count. In the fourth step (analysis), tissue is analyzed and results are stored in a data set. Using this method, two consecutive measurements of 303 slides showed an intraclass correlation of 0.99. In conclusion, slideToolkit provides a free, powerful and versatile collection of tools for automated feature analysis of whole slide images to create reproducible and meaningful phenotypic data sets.

## Introduction

Biobanking has become a significant corner stone in pathogenetic studies of multiple diseases and is an important resource for identifying mechanisms of many complex diseases. [1] It is evident that adequate and reproducible histological characterization of large amounts of collected of tissue is key, especially when used for association studies, such as genome wide association studies. In our Athero-Express biobank study, for instance, over 3000 patients have been included, which has resulted in >20.000 immunohistochemically stained cross-sectional slides using different types of antibodies that call for sufficient and consistent phenotyping. For immunohistochemical staining, antibodies are visualized by a chromogenic substrate, such as DAB (brown), 3-AEC (red) or a fluorescent dye. [2] To visualize the remaining tissue a hematoxylin (blue) counter stain is often applied. Until recently, we applied manual semi-quantitative scoring methods or case-by-case quantitative scoring of immunohistochemically stained cross-sections. However, manual or case-by-case phenotyping of histological slides is a time-consuming process liable to observer variability, and therefore, fast, unbiased and reproducible computerized phenotyping is indispensable. For many years, interactive morphometric techniques on live video images [3] and image analysis on sampled digital [4] have been applied, which has improved reproducibility, but this was still time consuming. A computer-aided method (analySIS FIVE, Olympos soft imaging solutions) to score inflammatory cells and smooth muscle cells quantitatively was previously implemented to improve reproducibility that indeed performed well. [5] However, this method requires the user to manually set color thresholds for the positively stained areas within subjectively selected regions of interest.

However, slides can now be completely scanned at high magnification in minutes with currently available slide scanners and stored as digital images, also known as whole slide images (WSI) or digital slides. After scanning, these slides can be viewed and analyzed on a computer. Automated quantification methods for analyzing WSIs are available. Generally, two methods exist: measuring surface area of a staining pattern, or identifying specific stained structures, like a cell, using advanced image analysis software. The first method, measuring surface area using a specific stain, is a fast approach that allows the measurement of stained areas in WSIs. Unfortunately, this method has a tendency of measuring 'false positive' or 'false negative' areas since only a range of colors are measured independent of morphological structures, such as individual cells. The other method of quantifying WSIs is by identifying each individual cell or blob using image analysis software. Unfortunately, the latter method can only handle relative small images (due to current computer hardware limitations) and works best with uncluttered cells and high-contrast stains (preferably fluorescence).

We here describe the validation of the slideToolkit, which is a collection of open-source libraries and scripts that handle each step of WSI analysis. Our goal was to create a fast method to handle large quantities of low contrast stained WSIs using advanced cell detecting algorithms. In this paper, we illustrate the power of slideToolkit for quantitation of CD3 stained cells using samples of our vascular biobanks.

## Methods

### Aneurysm-Express biobank

The Aneurysm-Express study (which is a derivative of the previously mentioned Athero-Express study) is a longitudinal biobank study that includes aneurysm tissue from all patients undergoing open surgical abdominal aortic aneurysm (AAA) repair in two Dutch hospitals. [6] The indications for open AAA repair were based on current guidelines. [7] The medical ethics committees of both hospitals approved the study, and all participants provided written informed consent. In short, during elective open AAA repair a full-thickness specimen of the ventral aneurysm wall was collected and is then transported to the lab where 5 mm segments were cut. The middle segment was fixed in 4% formaldehyde and embedded in paraffin for histological analyses; other segments were snap-frozen using liquid nitrogen and stored in −80°C freezers for future use. For histological analysis, consecutive sections were stained following standard procedures with hematoxylin and eosin (H&E), elastin Von Giessen (EvG), Sirius red, Von Willebrand factor, as well as immunohistochemistry (developed with diaminobenzidine (DAB)) for macrophages (CD68), T-lymphocytes (CD3) and B-lymphocytes (CD20) antigens. All slides were then archived in slide-cabinets.

To obtain a representative sample of our biobank, we retrieved the (arbitrarily chosen) CD3 stained AAA slides from our archive. These slides were obtained from 303 patients and routinely stained between 2005 and 2013. The selected samples were then retrieved from our archive, manually cleaned with alcohol and prepared for scanning.

### Ethics Statement

The Aneurysm-Express study was approved by the institutional review boards of both participating hospitals (University Medical Center Utrecht, Utrecht, The Netherlands, and St. Antonius Hospital, Nieuwegein, The Netherlands) and patients gave written informed consent.

### slideToolkit

**Figure 1:**
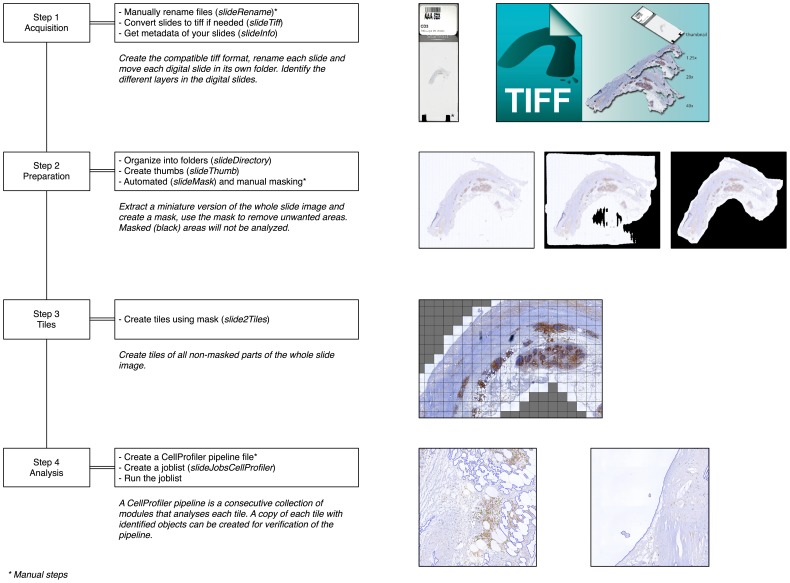
The four step slideToolkit workflow. An overview of the slideToolkit workflow with a summary and illustration for each step.

The slideToolkit is a collection of open-source scripts to handle each step from digital slide to the storage of your results. For a complete overview of all the tools, and there function in the slideToolkit, see table 1. An overview of the dependent libraries are listed in table 2. The toolkit is developed for modern (2014) personal computers (running *nix system [Linux, OS X, Unix]) and high-performance computing (HPC) systems. A common slideToolkit workflow consists of four consecutive steps. In the first step, “acquisition”, WSIs are collected and converted to TIFF files. In the second step, “preparation”, all the required files are created and organized. The third step, “tiles”, creates multiple manageable tiles to count. The fourth step, “analysis”, is the actual tissue analysis and saves the results in a meaningful data set. These steps are schematically depicted in figure 1. We present the slideToolkit as an open-source github repository (https://github.com/bglnelissen/slideToolkit).

**Table 1:** Tools available in the slideToolkit.

slideToolkit tools			
tool	Step	Function	Dependencies
**slideConvert**	Acquisition	Converts a whole slide image file to TIFF format	bfconvert, convert
**slideRename**	Acquisition	Batch rename slides	convert, display, identify, parallel, perl, tiffinfo
**slideInfo**	Acquisition	Fetch slide metadata (resolution, dates, etc)	awk, identify, openslide-show-properties, perl, tiffinfo
**slideDirectory**	Preparation	Create a staging directorie per slide	parallel
**slideThumb**	Preparation	Create slide thumbnail, including label	convert, parallel, perl
**slideMask**	Preparation	Create scaled mask and macro from a virtual slide	convert, parallel
**slide2Tiles**	Tiles	Cut virtual slide into tiles	convert, identify, openslide-write-png, parallel, perl
**slideJobsCellProfiler**	Analysis	Create a qsub joblist for the HPC to analyse all tiles	cellprofiler, convert, parallel
**slideSQLheader**	Analysis	Copy column headers from SQL file to CSV file	perl

**For each tool a accompanying help text can be found by running the tool followed by the —help flag (e.g. slideMask –help).**

Different tools are used in different steps. Most tools depend on other libraries and software packages.

**Table 2:** SlideToolkit software dependencies, licenses and project websites.

SlideToolkit dependencies		
Software	License	URL
bftools	GNU Copyleft ^a^	http://www.gnu.org/copyleft/
CellProfiler	GNU GPL v2 ^a^	www.cellprofiler.org
GNU Bash	GNU GPL v3 ^a^	www.gnu.org/s/bash/
GNU Parallel	GNU GPL v3 ^a^	www.gnu.org/s/parallel/
ImageMagick	ImageMagick License ^c^	www.imagemagick.org
Libitff	BSD-like licence ^b^	www.remotesensing.org/libtiff/
Openslide	GNU LGPL v2.1 ^a^	openslide.org
Perl	GNU GPL v1 ^a^	www.perl.org

**a:** www.gnu.org/licenses/

**b:** www.remotesensing.org/libtiff/

**c:** www.imagemagick.org/script/license.php

Copies of the different licenses can be found on the associated websites.

### Slide scanning

**Figure 2:**
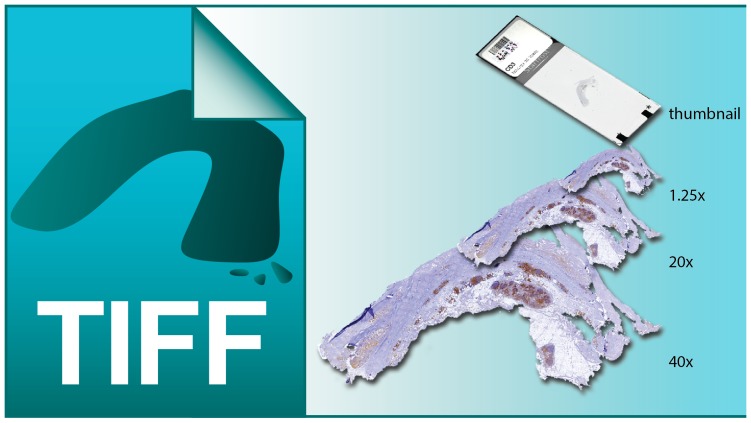
A visualisation of a multi-page pyramid TIFF file. This illustration shows a TIFF file with 4 layers (thumbnail, 1.25x, 20x, 40x), digital slides stored as TIFF files often contain up to 11 or more layers.

All slides were scanned using a Roche iScanHT whole slide scanner at 40x and digitally stored as a multi-page pyramid TIFF file (example in figure 2) containing separate layers (i.e. scanned tissue at magnifications of 40x, 20x and 1.25x, and a thumbnail image of each slide). The slideInfo command revealed that they consisted of 10 layers of different magnifications (ranging from 00.078x until 40x). Each layer was stored in JPEG format with a 90% compression level. Just before archiving, each digital slide was renamed manually using the ‘slideRename’ tool from the slideToolset as '*studynumber*.*stain*.tif' (e.g. AAA100.CD3.tif)

#### Step 1 – Acquisition

Most slide scanners are, in addition to their own proprietary format, capable of storing the digital slides in pyramid TIFF files. The slideToolkit uses the Bio-Formats library to convert other microscopy formats (Bio-Formats supports over 120 different file formats, www.openmicroscopy.org) into the compatible pyramid TIFF format if needed. TIFF is a tag-based file format for raster images. A TIFF file can hold multiple images in a single file, this is known as a multi-layered TIFF. The term "Pyramid TIFF" is used to describe a multi-layered TIFF file that wraps a sequence of raster images that each represents the same image at increasing resolutions (figure 2). The different layers contain, among others, the slide label and multiple enlargements of the tissue on the slide.

To read WSIs, the open-source libTIFF libraries and the OpenSlide libraries are used. These libraries are also applied to extract metadata (e.g. scan time, magnification and image compression) of the scanned slides. For image processing we use ImageMagick (ImageMagick 6.8.7-0 2013-10-16 Q16 http://www.imagemagick.org). ImageMagick is a command-line image manipulation tool that is fast, highly adjustable and capable of handling big pyramid TIFF files. Descriptive information about the slide is stored as metadata and contains, for example, pixels per micrometer, presence of different layers, and scan date.

#### Step 2 – Preparation

In the following steps multiple output files for each slide are created. For each digital slide, a staging directory is constructed in which the slide, and all output data concerning the slide are stored. In digital image manipulation, a mask defines what part of the image will be analyzed and what part will be hidden. Usually a mask can be defined as black (hidden) or white (not hidden). The slideToolkit creates a mask using convert (ImageMagick) and a miniature version of the WSI (in our example this is layer 6 of the multi layered TIFF). At first, the image is blurred, this will remove dust and speckles. Now, the white background is identified using a fuzzy, non-stringent selection and then background is replaced with black. Settings for blur and fuzziness can be found and changed in the slideMask tool. Generated masks can be adjusted manually in an image editor of choice (such as the freely available GNU Image Manipulation Program; GIMP (http://www.gimp.org)). Sometimes this is necessary to remove unwanted areas on the WSI (like marker stripes or air bubbles under the coverslip). For our analysis, only non-tissue parts of the WSIs were masked by BN (figure 1 - step 2).

#### Step 3 – Tiles

Image analysis of memory intensive, whole 20x representations of the digitized slides is currently impossible due to hardware and software limitations. The goal of this step is to create multiple smaller images (i.e. tiles) from the 20x WSI. An upscaled version of the mask is placed over the 20x WSI (in our example this is layer 3 of the multi layered TIFF). Image manipulation on 20x sized WSIs requires large amounts of computer RAM. To make it possible for computers without sufficient RAM to handle these files, the slideToolkit uses a memory-mapped disk file of the program memory. Using disk mapped memory files (ImageMagick.mpc files), the slideToolkit can efficiently extract all tiles. Without a mask, a faster and more memory efficient method is used.

#### Step 4 – Analysis

At this step, multiple tiles containing tissue data have been made, and the different objects in this tissue will be identified. CellProfiler is designed to quantitatively measure phenotypes from thousands of images automatically without training in computer vision or programming. CellProfiler can run using a graphical user interface (GUI) or a command-line interface (CLI). Using the CellProfiler's GUI, different algorithms for image analysis are available as individual modules that can be modified and placed in sequential order to form a pipeline. Such a pipeline can be used to identify and measure biological objects and features in images. Pipelines can be stored and reused in future projects. A pipeline for CD3 analysis using the CellProfiler GUI was created. The CLI was used to run the pipeline for actual image analysis. An illustrated example on how to create pipelines in CellProfiler is described by Vokes and Carpenter [8].

### Pipeline

**Figure 3:**
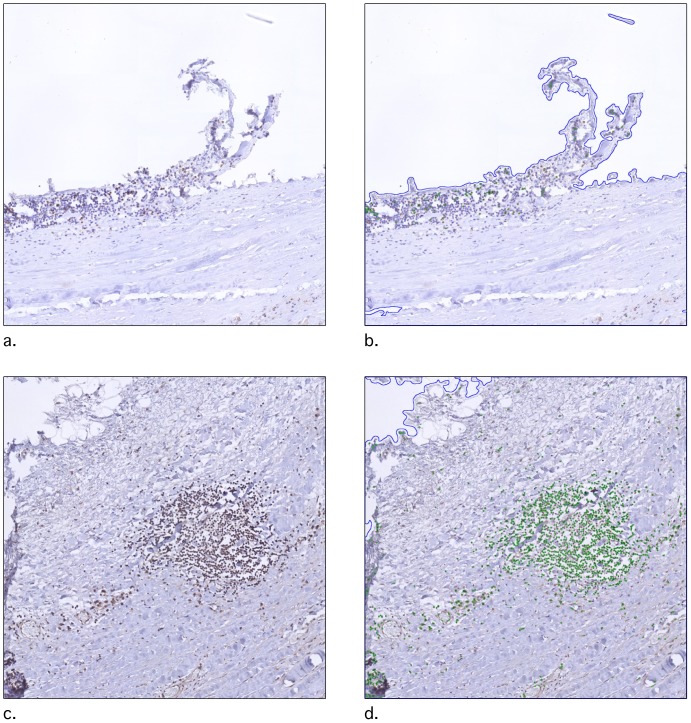
An example of object identification using the pipeline. a. and c. are original tiles, b. and d. are the same tiles with oulines of the identified objects. Blue lines outline the areas identified as tissue, green lines outline areas identified as DAB positive nuclei. These images are created using the 'SaveImages' module in CellProfiler.

We created a pipeline to assess the tissue surface area and the number of positively stained nuclei. Tissue was defined using the gray-scale version of the image, applying a Gaussian blur with a 20 pixels diameter, and inverting the resulting image (using the 'ColorToGray', 'Smooth' and 'ImageMath' modules respectively). Tissue was then identified as objects using the 'IdentifyPrimairyObjects'. We selected only objects larger than 40 pixels and with an absolute manual threshold of 0.03. Positively stained nuclei were identified as follows. The ‘UnmixColors’ module extracted the brown DAB color as a separate grayscale channel. In this DAB channel, nuclei were identified using the 'IdentifyPrimaryObjects' module. Positive nuclei were defined as objects with diameters between 8 and 26 pixels. Objects outside this range were discarded. An ‘Otsu Global’ with a ‘three classes’ threshold was used with pixels in the middle intensity class assigned to the foreground. Entropy was minimized. The threshold correction factor for pixels was entered as ‘1.3’, the lower and upper bounds on the threshold were ‘0.5’ and ‘1.0’, respectively. Clumped objects were distinguished by intensity. Occupied area for the 'Tissue' and the 'DAB' objects were measured using the 'MeasureImageAreaOccupied' module and exported to a.csv data file. To verify identified objects, an image was saved with an overlay of each object using the 'OverlayOutlines' and the 'SaveImages' modules (blue outline for tissue, green outline for DAB positive nuclei, figure 3).

We store CellProfiler measurements, like cell count, cell position, tissue surface area and other information in a database file (e.g. MySQL or.csv). Measurements can then be gathered and further analyzed using preferred statistical software, like R.

### Processing

For some steps, we ran the slideToolkit on local hardware (MacMini with 2 GHz i7 and 16 GB RAM). Other steps ran on a high-performance cluster (HPC) (8x Intel(R) Xeon(R) CPU E5-2630 0 @ 2.30 GHz, 38x Intel(R) Xeon(R) CPU E5-2640 0 @ 2.50 GHz, 11x Intel(R) Xeon(R) CPU E5-2630 v2 @ 2.60 GHz, all with 12 cores and 128 GB RAM per node) was used. Tools, wall-clock times and instructions can be found in table 3.

**Table 3:** Tools used, wall-clock times and instructions.

Tool	Step	N. slides	Wall-clock time	Command
**slideRename** *(Mac mini)*	Acquisition	303	2 hours ^a^	find. -iname "*tif" -exec slideRename —file = "{}" \;
**slideInfo** *(Mac mini)*	Acquisition	1	50 ms ^a^	slideInfo —file = "${HOME}/dir/DigitalSlide.tif"
**slideDirectory** *(Mac mini)*	Preparation	303	10 s ^a^	find. -iname "*tif" -exec slideDirectory —file = "{}" \;
**slideThumb** *(Mac mini)*	Preparation	303	4 m 23 s ^a^	find. -iname "*tif" | parallel slideThumb —file = "{}"
**slideMask** *(Mac mini)*	Preparation	303	5 m 39 s ^a^	find. -iname "*tif" | parallel slideMask —file = "{}"
**slideMask** *(HPC)*	Preparation	303	9.88 s [7.53–12.23] ^b^	qsub -pe threaded 1 slideMask —file = "/dir/DigitalSlide/DigitalSlide.tif"
**slide2Tiles** *(HPC)*	Tiles	303	6 m 36 s [322 s–1193 s] ^b^	qsub -pe threaded 3 slide2Tiles —file = "/dir/DigitalSlide/DigitalSlide.tif" —mask = "/dir/DigitalSlide/DigitalSlide.mask.png"
**slideJobsCellProfiler** *(HPC)*	Analysis	302	2 m 57 s [97 s–1934 s] ^b^	qsub -pe threaded 3 cellprofiler -i "/dir/DigitalSlide/DigitalSlide/tiles" -p "/dir/pipeline.cp"

**Wall-clock times are measured using GNU-time.**

**a: wall-clock time is calculated in total.**

**B: wall-clock time is calculated per digital slide (median, 1st–3rd quartile).**

**Mac mini: 2.0 GHz i7, 16 GB RAM (capable of 8 threads).**

**HPC: (8x Intel(R) Xeon(R) CPU E5-2630 0 @ 2.30 GHz, 38x Intel(R) Xeon(R) CPU E5-2640 0 @ 2.50 GHz, 11x Intel(R) Xeon(R) CPU E5-2630 v2 @ 2.60 GHz, all with 12 cores and 128 GB RAM per node), ‘-pe threaded 3’ represents three threads and 45 GB RAM (for each thread an additional 15 GB becomes available).**

One sample failed in the 'Tiles' step. Total elapsed wall-clock times are obtained using the GNU-time utility.

### Outcome

The 303 slides were scanned in two runs at 40x, using the Roche iScanHT slide scanner. From these 606 digital slides, masks were created, reviewed and adjusted. The number of CD3 positive cells per tissue area for each digital slide were compared between two runs. With the exception of the CellProfiler pipeline, all steps were unique for each 606 slides. Measurements were stored in.csv files. We used R (CRAN R version 3.0.2) to collect, concatenate and analyze the.csv files. Baseline characteristics of these measurements can be found in table 4.

**Table 4:** Digital slide file characteristics from Run 1.

File characteristics	
**Number of digital slides**	303
**Digital slide file size (median [IQR])**	774.00 [441.60–1297.00] MB
**Whole slide image x-axis dimension (median [IQR])**	33250 [25120–42520] pixels
**Whole slide image y-axis dimension (median [IQR])**	44880 [31110–62300] pixels
**Tiles per whole slide image (mean)**	208
**Tile file size (median [IQR])**	3.05 [1.72–5.27] MB

1 MB  = 1024*1024 bytes.

For each slide, measurements per tile were combined. Nuclei per area were defined as the number of CD3 positive cells per tissue area. Variability between measurements was determined by the difference in mean nuclei density per digital slide. The variability was analyzed by calculating intraclass correlation coefficient (ICC) using a two-way mixed single measure's model. In addition to ICC, Bland-Altman plots [9] were made to visualize the amount of agreements between the continuous scorings using the ggplot2 (version 0.9.3.1) [10] and psych (version 1.4.5) [11] packages in R statistics. Results of the raw CellProfiler measurements and companion R script (in which the ICC and Bland and Altman plots are calculated) can be found as supplemental data.

## Results

### Acquisition, Preparation & Tiles

We collected a total of 303 unique digital slides containing CD3 stained AAA tissue. File characteristics can be found in table 3. Each mask file was then visually checked using Adobe Photoshop CS6 (version 13.1.2) and adjusted if non-tissue was in the image (like the shade of coverslip-border or markings on the slide). Using these masks, tiles were created (using slide2Tiles) from the 20x layer of each digital slide and stored in the slide staging directory and analyzed by CellProfiler. A visualization of identified layers can be found in figure 2.

### Analyse & Data

One digital slide failed the conversion to tiles in both runs for unknown reasons. Tile analysis of the remaining 302 slides was done using CellProfiler (2.1.0.Beta_2a.linux/20131205152921). A CellProfiler pipeline was created by BN using multiple random tiles. Modules used in this pipeline are, among other, UnmixColors (to extract the DAB and the background stain), IdentifyPrimaryObject (to find nuclei within the positive DAB areas), OverlayOutlines (to visually check if object detection was correct) and ExportToSpreadsheet (to save the measurements data to file) (**Code S2**). Baseline characteristics of the two replicate measurements of 302 digital slides can be found in table 4. Wall-clock times of measurements done on the HPC are per slide, as the cumulative times would greatly depend on the availability of threats on the HPC.

The collected 303 digital slides used 290.63 GB of disk space. 1.22 TB of disk space was needed to store every file for our analysis. In total, 134976 tiles were analyzed. More runtime and file characteristics can be found in tables 3 and 4 respectively.

**Figure 4:**
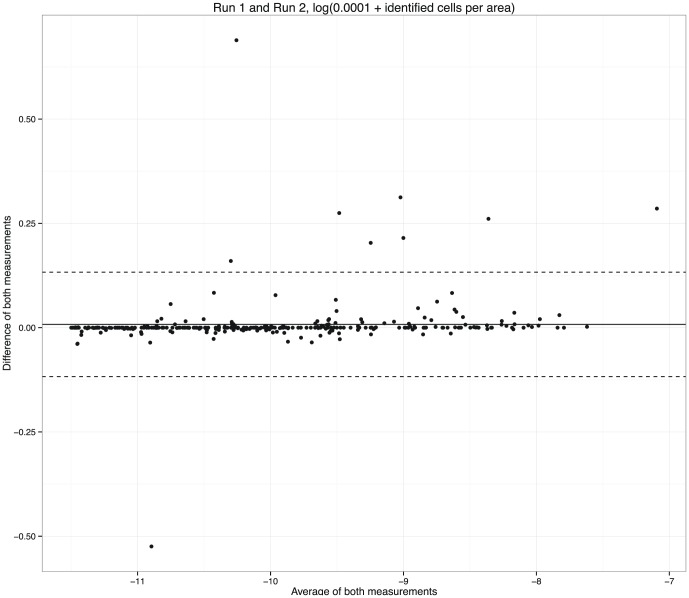
Bland and Altman plot of both measurements of number identified cells per area (Run1 and Run2). Measurements were log transformed, log(0.0001+ identified cells per area). The intraclass correlation coefficient (ICC) using two-way mixed single measures was 0.99.

**Figure 5:**
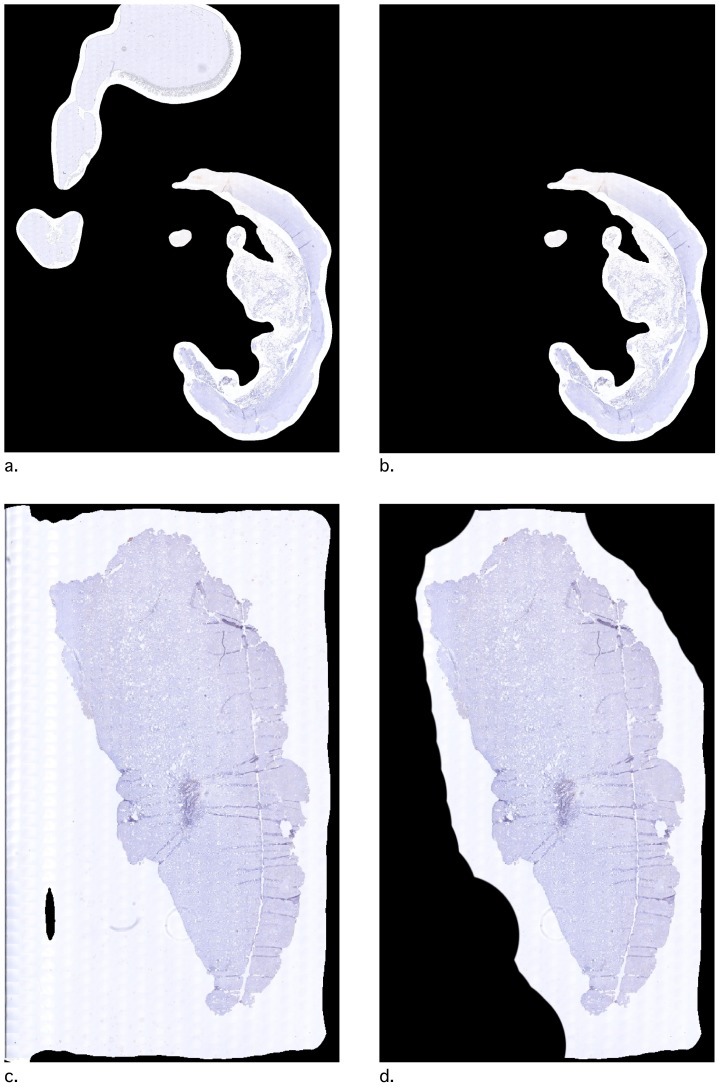
An example of discrepancy in mask formation. An air bubble was not masked in a., this same bubble was masked in b. The shadow of the coverslip (on the left side of the image) was not masked in c., this same shadow was masked in d. Automatically created masks should be checked manually to avoid unexpected results.

Variability between measurements was determined by the difference in mean nuclei density. The intraclass correlation coefficient (ICC) using two-way mixed single measures was 0.99. Bland and Altman plots to visualize the variability are depicted in figure 4. Outliers in this plot (differences of both measurements >0.50 or <0.50) are manually checked. This revealed that a discrepancy in masks caused these outliers. Figure 5 illustrates this difference; one mask contained air bubbles, and one mask contained a shadow artifact from the coverslip.

## Discussion

Biobanks become increasingly popular and the demand for adequate and reproducible histological characterization of large amounts of WSIs is increasing. For research purposes, manual assessment of a determinant in histologic slides is time-consuming and the reproducibility is not optimal, which is why computerized quantification of whole histological slides is preferred.

This study introduces the slideToolkit; a collection of open-source libraries and scripts which was developed to analyze WSIs. A slideToolkit workflow consists of four consecutive steps. In the first step, acquisition, digital slides are collected and converted to TIFF files. In the second step, preparation, files are organized in folders. In the third step, tiles, WSIs are divided into easy to handle tiles. The fourth step, (analysis), previously created tiles are analyzed and results are stored using CellProfiler. CellProfiler makes it easy to use advanced image analysis algorithms, and we demonstrate that we can reliably handle common DAB stains. A modern computer can do the process of slide analysis using our toolkit, but large quantities of WSIs are analyzed much faster using an HPC. An intraobserver ICC of 0.99 on 303 analyzed slides shows that WSIs can be accurately and reproducibly analyzed using the slideToolkit.

### Image quality

Bad images yield bad data. The quality of digital slides depends on many things. First, the histological slides need to be of sufficient quality. During fabrication, histologic slides must be produced using a uniform and strict protocol. Artifacts can occur that influence the sample quality due to improper processing, sectioning, staining and mounting of the tissue. [8], [12] Second, tissue on the slide is never completely flat. Most slide scanners, such as the one used in this study, automatically determine multiple focus points before scanning. Poorly selected focus points can result in blurred images and make it harder for the analysis software to detect objects. [13] Also, the slide scanner and its setup should be constant for every sample, including parameters like zoom levels, exposure times, shutter speed, focus, lighting conditions, and sensor sensitivity [8].

Furthermore, image compression is often used when storing large images. Image quality can be stored as lossy and lossless. Using a lossy compression, 'unnecessary' bits of information are discarded to reduce file size but this can cause artifacts in the image. In contrast, lossless compression does not discard visual information. Commonly used lossy image compression is JPEG and an example of a lossless compression is PNG. High-quality lossy compression can be sufficient for image processing, [14] but lossless compression is recommended. [8] We used 90% lossy compression (JPEG format) for the 20x WSIs which was sufficient for our analysis; lower quality settings were not tested. The slideToolkit uses lossless PNG compression when it extracts image components, so there is no further loss of image quality.

Masking can aid to exclude parts of the image from being processed. However, bad masks can result in unexpected measurements. Our results show a difference in measurements greater than 0.50 in two samples (figure 4). Retrospectively we learned that bad masking caused these outliers; one mask contained air bubbles, and one mask contained a shadow artifact from the coverslip (figure 5). A better manual check of these masks could have prevented such difference in measurements.

### Large quantities of slides versus speed

Adequate and reproducible histological characterization of large amounts of collected of tissue is key for, for example, the association with genetic data (e.g. large genome-wide association studies). Manually quantifying histologic slides is a difficult and time-consuming process, and the reproducibility is moderate to good. [5], [15] Computer aided analysis seems insurmountable when processing huge quantities of digital slides, but speed of the analysis depends on the architecture of the software. Various software tools have been developed for serial WSI processing, which can therefore only run as one thread (or task) at a time. Roughly speaking, using software built for serial processing, a faster computer would result in a more rapid analysis. However, there are other efficient methods to speed up analysis. The slideToolkit has been developed for parallel processing, which makes it possible to spread threads simultaneously over multiple computers. A professional computer cluster is not necessary. Using GNU parallel, a cluster of computers in a laboratory or an office will already result in enormous speed improvements. [16] The help menu within slideToolkit gives instructions on how to facilitate this.

### Software considerations

#### Open-source

We created the slideToolkit as open-source software following common development rules. [17], [18] These rules provide a fast way to build and improve software. It is promoted to release often and in early stages of development. Users will identify problems and new requirements in an early stage of development, and developers can fix them more quickly. Furthermore, providing the source code enables for more insight into what is happening. It also prevents other developers from re-inventing the wheel when encountering a similar problem. [18] SlideToolkit is released under the terms of the MIT license (http://en.wikipedia.org/wiki/MIT_License). In short, it implies that one can freely use the code, and that places almost no restrictions on what you can do with its source code. The software used by the slideToolkit is distributed under similar licenses (table 2).

#### Scripting

Most of the code is written in Bash (https://www.gnu.org/software/bash). Bash is a widely known scripting language and is a powerful way to streamline batch processes. Bash scripting makes it easy to exchange work flows between different computer systems.

### Image manipulation

To read digital slides, we use the open-source libTIFF libraries and the OpenSlide libraries. These libraries are also used to extract metadata (e.g. scan date, pixels per micrometer, magnification and image compression) of the scanned slides. For image processing, we use ImageMagick (ImageMagick 6.8.7-0 2013-10-16 Q16 http://www.imagemagick.org). ImageMagick is a command-line image manipulation tool that is fast, highly adjustable and capable of handling big pyramid TIFF files. When a mask is available, ImageMagick is used for the creation of tiles. The Openslide library is used when no mask is present.

### Image analysis

CellProfiler (www.cellprofiler.org) is used for the actual image analysis. CellProfiler makes use of modules that each performs an image-processing function, for example: unmixing colors into DAB and HE channels, object identification and measurement, storing measurements into a database. Most modules are highly configurable. A pipeline is a consecutive collection of modules to analyze images. The combination of different modules makes it possible to analyze even challenging images. Developing a working CellProfiler pipeline can be time consuming since it involves a lot of testing, and requires a certain homogeneity within the images you analyze. Variation of color intensities within WSIs can result in a consistent deviation of the outcomes. Pipelines can be reused and shared between computer systems and projects. The accuracy of the pipeline will determine the validity of the measurements.

In this paper we show an ICC of 0.99. This high correlation is found by looking at the number of identified cells per area. We have chosen for a specific cell type (nuclei of T-lymphocytes). One could argue that a small measurement error of a more present object (all nuclei) would result in a lower ICC. We have unpublished research were we find high ICCs in other tissue and for other measurements as well (ICC>0.90).

### Dependencies

The modular approach of the slideToolkit allows each tool to update independently without losing functionality of the toolkit.

### Future applications

Cloud based high-performance computing has become a popular way for scientists to do big analyses. The slideToolkit is primary developed for HPCs and is a powerful tool to take image analysis into the cloud.

CellProfiler creates the possibility to store the location of each identified object (e.g. a cell). When consecutive histological slides from tissue are stained for different antibodies, a virtual stack can be created and spatial information of the cells relative to each other can be analyzed.

### Shortcomings

During development we found that some slides contained undesired artifacts, like air-bubbles, pen markings or positive antibody control tissue. These artifacts can result in false measurements. To exclude these parts of the WSI we created the option to mask parts of the slide. Masking requires loading an uncompressed version of the WSI into the computer's memory and can require 60 GB or more. Masking is no issue for most HPCs, but can cause extremely slow performance on computers with insufficient RAM memory. Often, automatically created masks need to be altered to remove unwanted artifacts and this will influence reproducibility. Without the use of masks, undesired tiles can always be manually deleted before analysis.

### Code Availability

The slideToolkit is free software; you can redistribute it and/or modify it under the terms of the MIT license. The license and the source code for the slideToolkit can be found at: https://github.com/bglnelissen/slideToolkit. The present original paper is requested to be cited when using the slideToolkit to process data for publication.

## Conclusion

We present our open-source slideToolkit for manipulating and analysis of whole digital slides. The slideToolkit provides a free, powerful and versatile collection of tools for automated feature analysis of whole slide images to create reproducible and meaningful phenotypic data sets.

## Supporting Information

Code S1 slideToolkit.zip. An image of the slideToolkit source code. When using the code, you are encouraged to download the code from https://github.com/bglnelissen/slideToolkit.
(ZIP)

Code S2 pipeline.cp.zip. The CellProfiler pipeline used in this project.
(ZIP)

RawData S1 ICC and BlandAltman plot - measurements and r-script.zip. Measurements of Run1 and Run2, including the code for the calculation of the intraclass correlation coefficient (ICC) and the code for the Bland and Altman plot.
(ZIP)
